# Dimension Reduction of Multivariable Optical Emission Spectrometer Datasets for Industrial Plasma Processes

**DOI:** 10.3390/s140100052

**Published:** 2013-12-19

**Authors:** Jie Yang, Conor McArdle, Stephen Daniels

**Affiliations:** Energy Design Lab, Faculty of Engineering and Computing, Dublin City University, Glasnevin, Dublin 9, Ireland; E-Mails: mcardlec@eeng.dcu.ie (C.M.); stephen.daniels@dcu.ie (S.D.)

**Keywords:** dimension reduction, OES, plasma etching process, OES output pattern

## Abstract

A new data dimension-reduction method, called *Internal Information Redundancy Reduction* (IIRR), is proposed for application to Optical Emission Spectroscopy (OES) datasets obtained from industrial plasma processes. For example in a semiconductor manufacturing environment, real-time spectral emission data is potentially very useful for inferring information about critical process parameters such as wafer etch rates, however, the relationship between the spectral sensor data gathered over the duration of an etching process step and the target process output parameters is complex. OES sensor data has high dimensionality (fine wavelength resolution is required in spectral emission measurements in order to capture data on all chemical species involved in plasma reactions) and full spectrum samples are taken at frequent time points, so that dynamic process changes can be captured. To maximise the utility of the gathered dataset, it is essential that information redundancy is minimised, but with the important requirement that the resulting reduced dataset remains in a form that is amenable to direct interpretation of the physical process. To meet this requirement and to achieve a high reduction in dimension with little information loss, the IIRR method proposed in this paper operates directly in the original variable space, identifying peak wavelength emissions and the correlative relationships between them. A new statistic, *Mean Determination Ratio* (MDR), is proposed to quantify the information loss after dimension reduction and the effectiveness of IIRR is demonstrated using an actual semiconductor manufacturing dataset. As an example of the application of IIRR in process monitoring/control, we also show how etch rates can be accurately predicted from IIRR dimension-reduced spectral data.

## 1. Introduction

As indicated in recent International Technology Roadmap for Semiconductors reports [1], the future fabrication cost per-unit-area of integrated circuits (IC) will be decreased by moving to larger-diameter semiconductor wafers in the fabrication process, however, this move will require more sophisticated and precise process control mechanisms to ensure that process yields are preserved. Hence, driven by practical future manufacturing requirements, the design of process control mechanisms continues to be an active research topic in the IC manufacturing domain.

Plasma etching is a key processing method employed in IC fabrication steps. By first masking areas of the silicon wafer being processed, subsequent exposure to plasma yields the required etched features on the surface of the wafer. The process is fundamentally complex from a physical and engineering control perspective and sensitive to an array of process parameters [2]. As there is currently an incomplete understanding of the underling physics and chemistry to allow for pre-determined process control, etching processes are often developed empirically [3]. Critical to empirical control (and to the development of further fundamental understanding of the process) is the development of mechanisms for plasma monitoring by sensor data collection and analysis.

Generally, there are two types of plasma diagnostic sensors: intrusive sensors and non-intrusive sensors. One popular intrusive technology is the Langmuir probe [4], which is immersed directly into the plasma. Although direct measurements of targeted plasma parameters may be made, the direct immersion of the probe into the process environment results in changes in the temperature, density, and potential of the plasma and ultimately affects etching process results. Non-intrusive plasma process monitoring technologies include impedance monitoring [5], reflectometry sensing [6] and OES [7]. Due to the abundant information that can be extracted from the data and the direct (although complex) relationship of the data to the etching process, OES is widely applied to IC fabrication [7]. The richness of OES data is also a potential hindrance to effective interpretation and utility of the data. Of particular concern is data dimensionality. For example, a miniature Ocean Optics USB4000 fibre optic spectrometer, as used in the present work, provides intensity measurements of 2,048 wavelengths from 178.31 nm to 874.27 nm [8]. Full spectrum samples are typically taken every 0.7 s over typically 40 s of a dynamically changing process and datasets from hundreds of such process runs are taken for statistical analysis.

Previous research on OES measurements of plasma etching processes has largely focused on the use of OES data for particular target applications, for example, virtual metrology methods [9,10], automatic process end-point detection strategies [11,12], and system condition monitoring [13]. In this paper the focus is more directly on the fundamental dimensionality problem of OES data, so that such applications can be better facilitated. In the next section, our general approach to an appropriate dimension-reduction for the specific data type in question is introduced and our approach is distinguished from existing dimension-reduction methods. Section 3 describes our proposed *Internal Information Redundancy Reduction* (IIRR) method in detail. Section 4 demonstrates that little information content is lost when the method is applied to a dataset from a real semiconductor manufacturing environment. Additionally, practical problems relating to the particular spectroscopy data are addressed, namely data pre-processing steps to deal with sensor output saturation and data time-stamp uncertainty. As an example of the application of IIRR in process monitoring/control, we also show how etch rates can be accurately predicted from IIRR dimension-reduced spectral data. Finally, Section 5 gives our conclusions and future work ideas. Abbreviations used in the remainder of this paper are listed in Table 1.

## 2. Motivation for Approach to OES Dimension Reduction

Our overall approach to the design of an effective dimension-reduction method for OES data is guided by the following factors: (i) at a fundamental level, emission spectra from chemical species in a plasma are composed of emissions at discrete wavelengths only. Thus, we wish to isolate and work with only peak wavelength intensities in our spectral data, the assumption being that non-peak intensities represent only noise; (ii) as emission lines from each chemical species are highly correlated we expect considerable data redundancy within spectra; (iii) to maximize the utility of the dimension-reduced data, we wish to avoid transforming the data to an abstract variable space (as is common in many dimension-reduction methods), instead working directly with wavelength variables; (iv) as plasma processing is a dynamic process, it is important to preserve time domain information, that is, our focus is on dimension reduction in the wavelength domain only.

From a plasma-etching viewpoint, there has been little focus on dimension and redundancy reduction of the OES dataset per se. Most previous research has been focused on application of the dataset (e.g., for process fault detection) where dimension reduction is used as a data pre-processing step but is not the focus itself. In [14], principal component analysis (PCA) (in conjunction with a hidden Markov model) is used for process end-point detection in plasma etching processes. In [15], a weighted PCA method is proposed for fault detection and classification in plasma etching. Besides OES data, other plasma diagnostic datasets were also used such as chamber impedance measurements and gas flow measurements. In order to reduce data dimensions, the original data is replaced by several summary statistics, such as averages, standard deviations, maxima, minima. In [16], Sparse PCA (SPCA) is used to select signature OES variables. In [17], Partial Least Squares (PLS), support vector machines, and rules ensemble methods are compared with each other for process yield prediction. Dimensionality of the input data is reduced using PLS and rules ensemble within the prediction process.

A general feature of these previous applications of dimension reduction of OES data is that generic methods (e.g., PCA, SPCA, or use of summary statistics) are applied directly to the full set of input wavelength variables, without regard to the specific nature of the dataset and these methods can have difficulty in finally isolating important variables in the original variable space. For example, it is not possible to trace back to individual wavelength measurements at a certain time point when only summary statistics are the output of the method [15]. In PCA-based methods, every Principal Component (PC) is a linear combination of all original variables. This is a problem if quantification of the contribution by each variable to certain PCs is required [18]. SPCA is a possible solution to this problem [19], but the grouping effect (equal weights tend to be given to highly correlated variables) is a weakness, leading to difficulty in final variable selection [16].

Other general dimension-reduction methods also have disadvantages for direct application to the problem at hand. Ensemble methods have been shown to be successful in identifying important variables in the original space [20], however ensemble learning methods (e.g., boosting, bagging [21], rules ensembles [20]) need to be supervised by knowledge of output variables, which in our case would be actual etch-rate measurements, which are normally not available. Other supervised learning methods are similarly unsuitable in the current context. Factor Analysis (FA) [22], projection pursuit [23], Artificial Neural Networks (ANN), and Independent Component Analysis (ICA) all have their own particular issues. In [24], a number of problems are highlighted for the FA method, where it is often possible to extract too few or too many factors and factor stability can be a concern. For projection pursuit [23], high computational intensity is a disadvantage [25]. Compared with PCA, ANN gave a better dimension reduction result in [26], however, ANN can suffer from relevantly high computational load, the over-fitting problem, and the empirical nature of model development [27]. ICA has a similar information transformation strategy as PCA and FA but can have difficulty in determining component number and component order [28]. Both problems will lead to a high computational cost and difficulty for further interpretation of components.

Based on the above mentioned difficulties in directly applying general dimension-reduction methods in our specific domain, we propose in this paper a new method, called *Internal Information Redundancy Reduction* (IIRR). A central feature of the method is the importance of peak values of the wavelength variables comprising the dataset, and consequently the opportunity to remove variables which do not exhibit significant peaking. Secondly, high correlation between certain groups of peak variables is known to exist, given the nature of the physical emission process. This helps us in designing our method to maximise redundancy reduction. We develop our method in the following sections, showing that a large reduction in the number of variables is possible with little information loss.

## 3. Dataset Description and Method Overview

A complete dataset of OES data is comprised of time-stamped spectral scans collected over multiple etching process runs. There are *N* process runs made and a spectral scan is taken at each time instance *t_j_*, *j* = 1, 2, …, *M*, during each run. Each spectral scan yields a set of wavelength intensity measurements {*I_k_*:*k* = 1, 2, …, *K*}. The *K* wavelengths measured { *λ_k_*:*k* = 1, 2, …, *K*} are equally spaced, typically across a range from *λ_1_* = 178.31 nm to *λ_k_* = 874.27 nm, with *K* = 2,048 total wavelength values. Process run durations typically span approximately 40 s with spectral scans taken approximately every 0.7 s. Thus the complete dataset is the set of *N* × *M* × *K* data points, which we denote by the vector:

(1) $$\begin{array}{cccc} \text{d}=\langle i,t_{j},I_{k}\rangle , & i=1,2,\ldots ,N, & j=1,2,\ldots ,M, & k=1,2,\ldots ,K \end{array}$$

We note here that the sensor employed in OES measurement can saturate its output value at certain wavelengths that are prominent in the process. A method to deal with de-saturation is described in Appendix 1. We additionally note that, in the raw OES input data, time stamps from one process run to another may not always exactly align (the time between scans can vary during each process run). Also, the number of time points recorded for each process run is not necessarily the same. Thus, we must re-align/normalize the data in time before it is inputted to the IIRR procedure. This is done using the method described in Appendix 2 to yield data with an equal number of time points *M* for all process runs, with time points fixed to standard time instances *t_j_*, *j* = 1, 2, …, *M* (with *t_j_*–*t_j-1_* constant) for all process runs. We assume de-saturated and time-synchronized data in the above and further definitions and descriptions in this section.

Given the above data as input, our proposed method is a dimension-reduction method with three sub-steps:

(1) Absolute Peak Selection (APS),
(2) Iterative Ranking Process (IRP),
(3) Optimized Peak Selection (OPS).

**Figure 1.**
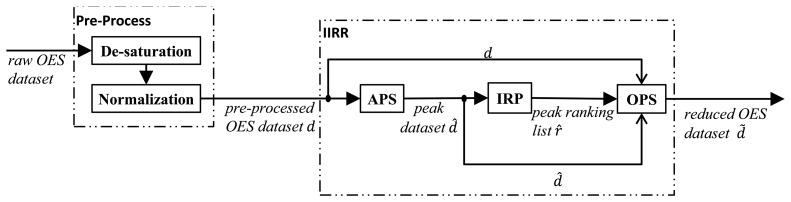
Workflow of IIRR.

Together they comprise the *Internal Information Redundancy Reduction* (IIRR) workflow, depicted in Figure 1. Firstly, APS identifies peak wavelength variables in the (de-saturated and normalized) input OES dataset ***d***. The output of APS for a time point *t_j_* is the set of wavelength indices for which peaks in wavelength intensity occur, at time point *t_j_*, during any of the *N* process runs. Formally, the output of APS is the collection of sets of peak wavelength indices, denoted:

(2) $$\hat{\text{d}}=\langle {\hat{d}}_{t_{1}},{\hat{d}}_{t_{2}},\ldots ,{\hat{d}}_{t_{M}}\rangle$$

Where *d̂_t_j__* = {*k* ∈ {1, 2, …, *K*}: *I_k_* is a peak wavelength intensity at time *t_j_* in at least one process run}. Next, the IRP algorithm takes each set of peak wavelengths *d̂_t_j__* and ranks each peak wavelength variable in the set based on how well it can be predicted from other peak intensity values in *d̂_t_j__*. Peaks that are poorly predicted by others are assumed to hold more significant information and are ranked more strongly. Repeating the procedure for each time point, the output of IRP is a dataset of the same dimensions as ***d̂*** containing wavelength indices in order of ranking, *i.e.*, a dataset ***r̂*** = 〈*r̂_t_*__1__, *r̂_t_*__1__, …, *r̂_t_M__*〉, where:

(3) $${\hat{r}}_{t_{j}}=\text{the sequence}\;\langle k_{1},k_{2},k_{3},\ldots \rangle \;\text{where}\;\text{rank}(k_{l-1})<\text{rank}(k_{l}),\forall k_{l},k_{l-1}\in {\hat{d}}_{t_{j}}$$

Finally, at each time point, the OPS algorithm calculates a measure of how well the first *r* top-ranked peak wavelength variables can predict the full set of original wavelength variables, at each time point *t_j_*. An aggregate statistical measure *Mean Determination Ratio* (MDR) is used to summarize the prediction quality for different *r* and a minimal value of *r* is determined under a constraint on the MDR value. The procedure is repeated at each time point and the final output of OPS is a time series of the minimized peak wavelength sets, denoted ***d̃***. The details of these three stages of the IIRR procedure are given in the subsections below, accompanied by basic results illustrating the properties of each stage. Results in Section 4 then show that the whole procedure results in a high level of data reduction for a sample OES dataset, without significant loss of predictive power of the reduced set of variables.

### 3.1. Absolute Peak Selection

Absolute Peak Selection (APS) is a simple method to identify wavelength intensity variables that are relatively higher in value than neighboring wavelength intensities, while accounting for noise in wavelength intensity measurements. The noise accompanying each wavelength intensity measure is represented as a mean bias value *B*, which is derived from the specifications of the spectrometer. In our case, the USB4000 Spectrometer signal-to-noise ratio is quoted as 300:1 at full signal [8] and the appropriate value for *B* is derived from this in Appendix 1. APS operates independently at each time point *t_j_* and, for each process sample, identifies a wavelength variable as a peak wavelength if its intensity is greater than the intensities of neighboring wavelengths plus the bias value. Let *d_i.tj_* be the set of wavelength intensity values {*I_k_*:*k* = 1, 2, …, *K*} measured at time point *t_j_* during process run *i*. Then peak wavelengths within this set are identified by the set:

(4) $${\hat{d}}_{i,t_{j}}=\{k:I_{k-1}+B<I_{k}<I_{k+1}+B,1<k<K\}.$$

Having found *d̂_i,tj_* for all process runs 1, 2, …, *N*, all peak wavelength variables are then aggregated as:

(5) $${\hat{d}}_{t_{j}}=\overset{N}{\underset{i}{\cup }}{\hat{d}}_{i,t_{j}}$$

Finally, iterating this procedure at each time point, the complete output of APS is ***d̂*** = 〈*d̂_t_*__1__, *d̂_t_*__1__, …, *d̂_t_M__*〉. We note that the rationale for this aggregation in the above equation is that the data gathered from each process run is an independent sample of an (ostensibly; fixed etching procedure, thus the process state should be similar for all samples at the same time point and so aggregation can achieve data reduction without significant loss of information content.

Based on our OES dataset from a semiconductor etching process, we have found APS reduces the original 2,048 wavelength variables to a relatively small number of peaks at each time point, ranging from 22 to 113 peaks (averaging ∼47.7 peaks). Over all time points, 178 distinct peak wavelengths are detected.

### 3.2. Iterative Ranking Process

The ultimate goal of the *Iterative Ranking Process* (IRP) and *Optimal Peak Selection* (OPS) methods is to identify which subset of the peak wavelength variables can best represent the remaining variables, so that the set of peak wavelengths can be reduced with minimal information loss. This is done by the ranking procedure of IRP followed by a selection from top-ranked variables performed by the OPS procedure (Section 3.3).

Each set of peak wavelength intensity samples *d̂_t_j__* is treated separately by IRP, as follows. For each wavelength intensity variable *I_k_*, *k ∈ d̂_t_j__*, an ordinary least squares linear regression is performed:

(6) $${\tilde{I}}_{k}=\beta_{k,1}I_{1},\beta_{k,2}I_{2}+\cdots +\beta_{k,k-1}I_{k-1}+\beta_{k,k+1}I_{k+1}+\cdots +\beta_{k,K}I_{K}+\varepsilon_{k}$$

Where *β_k,k^′^_* are the regression coefficients and *ε_k_* the error term. IRP then calculates the coefficient of determination (the R^2^ value) of the prediction *Ĩ_k_* of *I_k_*, which we denote by *R_k_*. The lowest ranked wavelength in *d̂_t_j__* is then identified as having the largest R^2^ value:

(7) $$\text{rank}(k)=\left|{\hat{d}}_{t_{j}}\right|\;\text{if}\;\underset{l\in {\hat{d}}_{t_{j}}}{\max}R_{l}=R_{k}$$

that is, the ranking number assigned to wavelength *k* is equal to the total number of peaks in *d̂_t_j__*. This wavelength *k* is then removed from the pool of peak wavelengths and the process is repeated on the new set *d̂_t_j__* –*k* to yield the next ranked wavelength (rank number |*d̂_t_j__*| – 1). The process repeats until only one wavelength remains in the pool, which is assigned the highest ranking (rank number 1). The complete output of the IRP process (for the time point *t_j_*) is then the ordered set of wavelength indices:

(8) $${\hat{r}}_{t_{j}}=\text{the sequence}\;\langle k_{1},k_{2},k_{3},\ldots \rangle \;\text{where}\;\text{rank}(k_{l-1})<\text{rank}(k_{l}),\forall k_{l},k_{l-1}\in \hat{d}t_{j}$$

This IRP process is repeated for each time point to yield the final output:

(9) $$\hat{\text{r}}=\langle {\hat{r}}_{t_{1}},{\hat{r}}_{t_{2}},\ldots ,{\hat{r}}_{t_{M}}\rangle .$$

The rationale for the method is that peaks that are removed from the pool early (low ranked peaks) can be well predicted by the remaining pool of peaks and so hold relatively less information. Peaks that remain in the pool are less correlated with others and are ranked higher. We note that in our IRP method, removing peaks and reiterating the evaluation with a decreasing pool size should improve the sensitivity of the ranking between peaks. Particularly, in very highly redundant datasets, a simpler method of ranking based only on a single evaluation of R^2^ over the full pool can yield all R^2^ values very close to 1, giving only a weak distinction between peaks.

**Figure 2.**
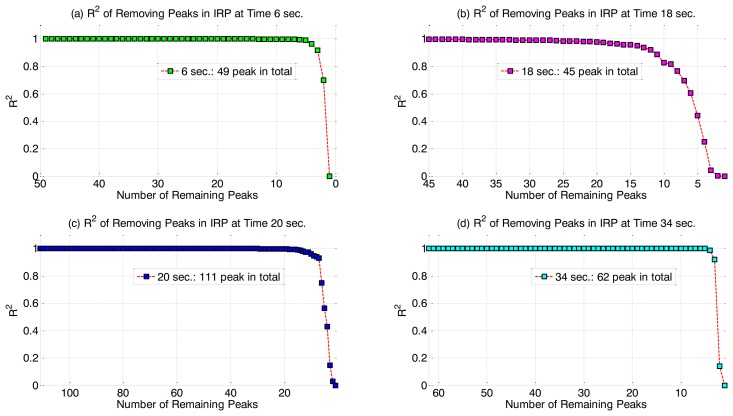
R^2^ values of regression of the remaining peaks in the pool on the peak to be removed in each IRP iteration (**a**) at time 6 s, (**b**) at time 18 s, (**c**) at time 20 s, and (**d**) at time 34 s, as a function of remaining number of peaks in pool.

We apply IRP to the APS output of the OES test dataset mentioned in Section 3.1. Figure 2 shows samples of the IRP output for four example time points during the etching process. The curves show the maximal coefficients of determination *R_k_* as the procedure iterates, that is, the R^2^ value of the peak to be removed from the pool at each iteration. At the start of the procedure the R^2^ values are very close to 1 and towards the end of the procedure, only the highest ranked peak variables, with lower R^2^ values, remain. In terms of identifying an opportunity for data reduction, it can be seen that only relatively few high rank peaks have lower R^2^ values. This general pattern was observed at all time points in the IRP output.

### 3.3. Optimal Peak Selection

Having ranked peak wavelength variables using IRP, the *Optimal Peak Selection* (OPS) procedure selects a top-ranked subset of the peak variables. The number of peaks in this subset is minimized under the constraint that the prediction of all wavelength variables by the subset of peak variables meets a specified prediction quality target. More formally, let *r̂_t_j__* be the ranked set of peak wavelength indices <*k_1_*, *k_2_*, *k_3_*, … >, for time point *t_j_*, and let *K'* be the size of this set. For each *r* ∈ {1, 2, …, *K'* }, the OPS procedure regresses wavelength intensity variable {*I_k_*:*k* = 1, 2, …, *K*} on the set of peak wavelength variables <*I_k1_*, *I_k2_*, …, *I_kr_*>, to yield prediction *Ĩ_k_*, and calculates how well *Ĩ_k_* predicts *I_k_* by way of the R^2^ value denoted *R_r,k_*. (Similarly to the IRP procedure, an ordinary least squares linear regression is used). An aggregate measure of how well the set of peak variables <*I_k1_*, *I_k2_*, …, *I_kr_*> predicts the full set of wavelength variables {*I_k_*:*k* = 1, 2, …, *K*} is calculated, by way of our *Mean Determination Ratio* (MDR) metric, defined as:

(10) $${\text{MDR}}_{r}≜\frac{\sum_{k=1}^{K}R_{r,k}}{K}.$$

Finally, an optimal value of *r*, denoted *ř_l_* is determined (as explained below), which selects the final reduced set of peak wavelength variables *d̃_t_j__* = 〈*Î_k_*__1__, *Î_k_*__1__, …, *Î_k_ř_j___*〉, for this time point *t_j_*.

**Figure 3.**
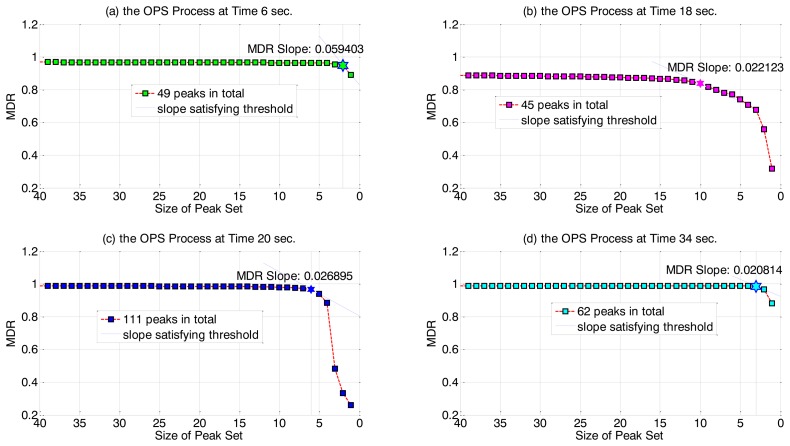
The MDR values for differently sized candidate peak sets (*r* values less than 40 shown) (**a**) at time 6 s, (**b**) at time 18 s, (**c**) at time 20 s, and (**d**) at time 34 s.

Empirically, we have found that as *r* is initially decreased from its maximum value of *K'*, the prediction quality (MDR value) remains at a high value and decreases very slowly. Eventually, as *r* approaches 0, the MDR begins to drop off quickly. (Figure 3 illustrates this pattern for our test OES dataset). Thus, we have chosen to use a threshold on the slope of MDR_r_ to determine the optimal value *ř_j_* that gives a small variable set but with still high MDR value, that is, in the OPS procedure *r* is decreased from its maximum value until:

(11) $$({\text{MDR}}_{r}-{\text{MDR}}_{r-1})>m_{\text{threshold}}$$

where *m_threshold_* is chosen to achieve a desired trade-off between prediction quality and the number of remaining peak variables. The above process is repeated for each time point to yield the final output of the IIRR procedure as ***d̃*** = 〈*d̃_t_*__1__, *d̃_t_*__2__, …, *d̃_t_M__*〉:

Figure 3 illustrates typical output of the OPS stage, at four example time points. We have again used our sample OES data (preprocessed by APS and IRP) and set an MDR slope threshold of 0.01 for the OPS selection criterion. Even at a small slope threshold it can be seen that only few peak variables remain in the final selected set and so good data reduction is achieved. Over all time points, *ř* values have been observed to be low, spanning the range of 1 to 10 with an average of 5.46 remaining peaks per time point (compared to 47.7 variables on average after only the APS stage).

## 4. Experimental Results and Discussion

In the previous section we have shown that, when applied to our test data set, the IIRR method can reduce the number of input variables by a large degree without a significant loss in prediction accuracy from the remaining variables to the full set of original input variables. To further validate the method, in this section we quantify the prediction quality of the reduced set of variables produced by IIRR when predicting an independent output variable, the etch rate. Although this measurement is not normally available from plasma etch process monitoring, for our particular test dataset of spectral data from a real semiconductor etching process, we have a corresponding final etch rate measurement for each process run. Our validation procedure is as a follows.

**Figure 4.**
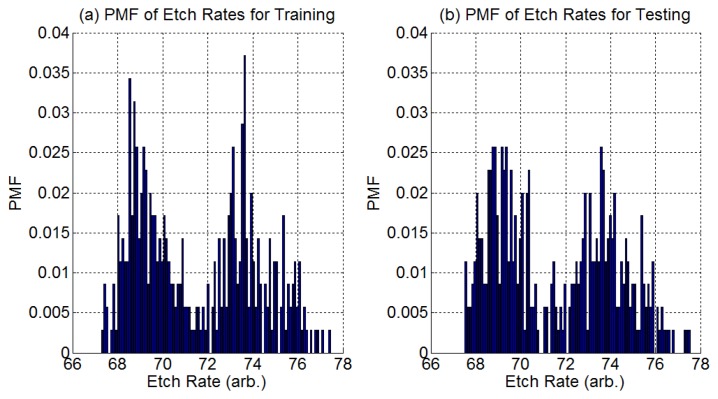
Estimated Probability Mass Function (PMF) of etch rates for (**a**) training group and (**b**) testing group.

We have 900 process samples (process runs) which we split equally into a training group and a testing group. A process sample contains the time series OES data for the process run plus one final etch rate measurement. The distribution of all etch rate samples in each group is shown in Figure 4.

The IIRR procedure is used to find a reduced set of wavelength measurements using only the training OES sample group. We note that the etch rate variable is not part of the IIRR training input, only the OES training samples.

**Figure 5.**
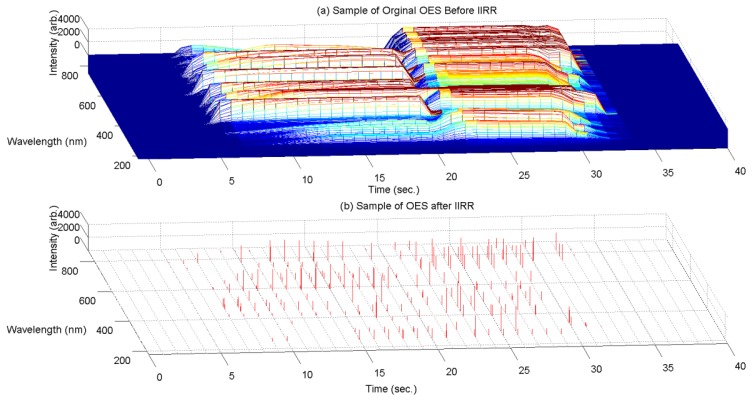
(**a**) Sample of original OES data and (**b**) a sample of the selected peaks after IIRR for a complete etching process.

A sample of the OES measurements before and after the IIRR process is shown in Figure 5, for a typical process run. It can be seen that data dimension is reduced significantly. Across all samples, less than 0.27% of the original 2,048 wavelength measurements remain after IIRR.

We next compare the prediction accuracy of the IIRR reduced dataset to the prediction accuracy when using the full set of OES data (we note that the full data set is first de-saturated and time normalized, see Appendixes 1 and 2), resulting in input data with 1,807 wavelength variables, of a possible 2,048, over 41 time points). In either case, wavelengths (or peak wavelengths) at each time point are treated as independent parameters for prediction. Three popular prediction methods are tested to provide a regression-independent result: multiple linear regression (MLR), PLS, and ANN. Table 2 summarizes the results. For comparison, also included in the table are predictions of the training data from itself.

**Figure 6.**
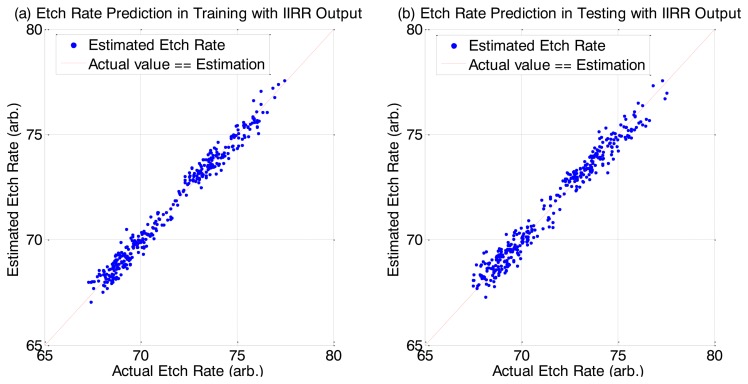
Individual etch rate predictions from the IIRR reduced dataset using PLS.

We can see very good R^2^ and MAPE scores for the predictions. Interestingly, for the prediction of the testing dataset, there is better prediction accuracy (for MLR and PLS cases) when using the IIRR reduced dataset compared to using the full dataset. We attribute this improvement to the noise reduction effect of IIRR. We additionally note that PLS achieves the best result. It has been noted previously, in [28], that PLS is a more suitable method than MLR when the data dimension is large and there is high redundancy. In addition to good overall R^2^ and MAPE values, there is very good prediction accuracy across all individual samples, as shown in the Figure 6.

## 5. Conclusions and Future Works

We have presented a new *Internal Information Redundancy Reduction* (IIRR) method for reducing the dimension of time series samples of OES measurements and, by use of real sample data from a plasma semiconductor process, have shown that the method can effectively reduce the number of wavelength intensity measurements required to accurately represent the data. As validation, we showed that prediction of an independent output variable (etch rate) can be done very effectively with the reduced set of variables, which comprise less than 0.27% of the original variables. In fact, prediction accuracy was slightly improved, compared to prediction with the full set of input variables.

We note that our IIRR operates in the original variable space, rather than a transformed variable space, which would make the method useful for OES analysis methods whose goal relates to physical interpretation of the data and process, for example in virtual metrology methods. We would also expect the method to be effective for application to high-dimensional spectral data from other processes, where the dataset represents a set of time series, each of which is an independent sample from the same fundamental process. Although the APS step of the algorithm is specific to OES datasets, the core method (IRP + OPS) could be expected to be effective for other (non-OES) high-dimensional time series datasets, where multiple independent samples of the same (repeatable) underlying process behaviour are available. However, we note a caveat here. As the IRP phase of the method ranks less correlated variables highly, there is a risk of biasing noise for inclusion in the final variable set. In our case, our interpretation of non-peak data as noise and its effective reduction/removal by APS avoids this scenario. For data from other processes, some similar insight to the nature of the noise and an effective noise reduction method would be required, so that a high level of data reduction can be achieved. On the other hand, as our IRP/OPS method is ‘internal’ in nature, not guided/biased by a chosen output variable(s), it is conservative in terms of attempting to distinguish unexplained variation from noise. As a stand-alone method of preparing a universal reduced OES dataset, that can be applied to prediction of multiple different output variables of interest, this may be useful.

Future work will investigate application of the method to other such data sets. Additionally, we will in future also consider how redundancy in the time domain can be reduced, which we have not considered in the present paper. In relation to our current OES plasma data, at least for certain periods of the process when it is less dynamic, the process is most likely over sampled and there is an opportunity for further reduction without significant loss of important time domain information.

## Figures and Tables

**Table 1.** Acronym table.

**Acronym**	**Definition**
ANN	Artificial Neural Network
APS	Absolute Peak Selection
FA	Factor Analysis
IC	Integrated Circuit
ICA	Independent Component Analysis
IIRR	Internal Information Redundancy Reduction
IRP	Iterative Ranking Process
MDR	Mean Determination Ratio
MAPE	Mean Absolute Percentage Error
MLR	Multiple Linear Regression
OES	Optical Emission Spectroscopy
OPS	Optimized Peak Selection
PC	Principal Component
PCA	Principal Component Analysis
PLS	Partial Least Square
PMF	Probability Mass Function
SPCA	Sparse Principal Component Analysis
SNR	Signal to Noise Ratio

**Table 2.** Etch rate prediction accuracy comparison between original (full) OES dataset and the IIRR reduced dataset (with *m_threshold_* = 0.01 in OPS procedure).

**Regression Method**	**Input Dataset (Total Number of Input Variables)**	**Training Prediction Accuracy**		**Testing Prediction Accuracy**	

		**R^2^**	**MAPE**	**R^2^**	**MAPE**
MLR	IIRR Reduced Dataset (224)	0.9930	0.0024	0.9430	0.0070
	Complete Dataset (2,048 × 41)	0.9944	0.0021	0.9329	0.0074

PLS	IIRR Reduced Dataset (224)	0.9802	0.0041	0.9705	0.0051
	Complete Dataset (2,048 × 41)	0.9805	0.0041	0.9676	0.0053

ANN	IIRR Reduced Dataset (224)	0.9710	0.0042	0.9049	0.0084
	Complete Dataset (2,048 × 41)	Input too large for computation			

**Figure A1.**
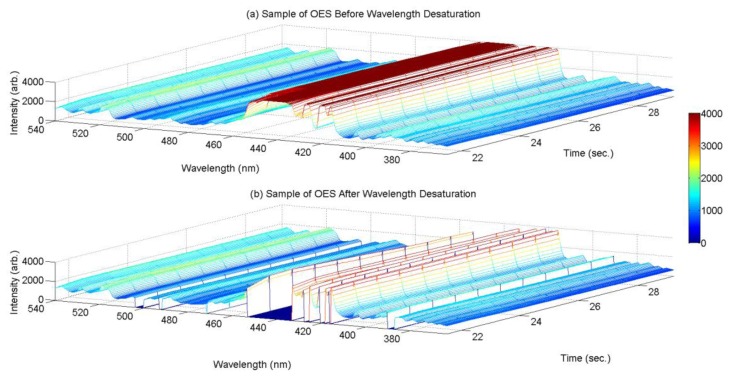
Sample of OES measurements (**a**) before and (**b**) after desaturation.

**Figure A2.**
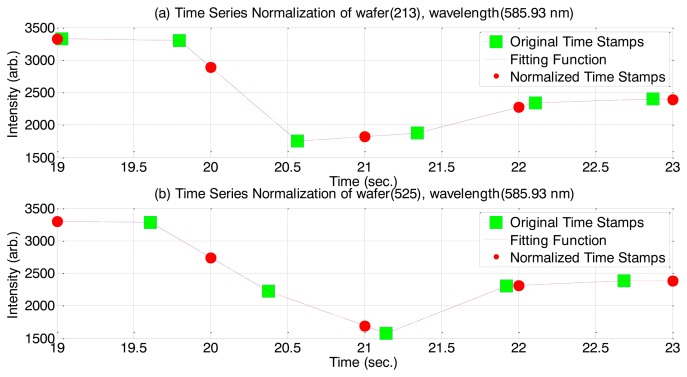
Original and normalized time series of wavelength (585.93 nm) from (**a**) sample 213 and (**b**) sample 525.

## Appendix I. Pre-Process: Wavelength Desaturation

Our particular OES test dataset presents a specific problem of saturated values in some wavelength intensity measurements, which we deal with before inputting our dataset to the IIRR procedure. Given the typical limited Signal-to-Noise Ratio (SNR) in OES measurement (in this case, the SNR is 300:1 at full signal [8]) it is judicious to set sensor gain to accept some saturation in measurement in return for increased measurement sensitivity for low intensity values. However, the resulting saturated values (see red plateaus in Figure A1a) should be removed from the dataset to avoid interpretation as correlation between the saturated values.

Our approach is simply to remove all wavelength variables at each time point that exhibit saturation, where our test for saturation is: for each wavelength *k*, is:

(12) $$I_{k}>I_{\text{max}}-B$$

for any sample, where *I_k_* is the intensity value under test, *I_max_* is the maximum optical intensity of the sensor and *B* is the sensor bias, estimated as:

(13) $$B=\frac{I_{\text{max}}}{\text{SNR}}$$

with an *SNR* of 300:1 at full signal for the spectrometer in [8]. Figure A1b shows the result of removal of the saturated intensity values. Over the full dataset, 241 of the 2,048 wavelengths are removed.

## Appendix II. Time Series Normalization

Each etching process run outputs a time series of spectral intensity scans, however, the sequence of timestamps from one process run to another is not necessarily identical. As the IIRR method needs to group all samples at a given time point during its data processing stages, the timestamps need to be aligned to a normalized time scale. The time between samples averages approximately 0.7 s and, over all process samples, the minimum final time stamp is 40.14 s. We set the normalized time scale to have 1 s intervals, with the final timestamp at 40 s. Having set the time scale, the values in each time series (process run) are transformed by linearly interpolating the wavelength intensity values between the points either side of exact 1 s intervals. The process is illustrated in Figure A2 below with two representative samples from the data.
